# Microstructural Properties and Pressure Distribution in Ultra-Short-Pulse Welds of Sapphire to Iron

**DOI:** 10.3390/nano16120737

**Published:** 2026-06-13

**Authors:** Lukas Günther, Anne Friedrich, Jens Ulrich Thomas, Thomas Müller, Dominique de Ligny

**Affiliations:** 1 Department Werkstoffwissenschaften, Institut für Glas und Keramik, Friedrich-Alexander-Universitat Erlangen-Nürnberg (FAU), Martensstraße 5, 91058 Erlangen, Germany; 2 SCHOTT AG, Hattenbergstraße 10, 55122 Mainz, Germany; 3 Analytical Mineralogy, Institute of Geosciences, Friedrich Schiller University Jena, Lessingstraße 14, 07743 Jena, Germany; 4 Geoscience Centre, Georg-August-Universität Göttingen, 37077 Göttingen, Germany

**Keywords:** welding, sapphire, ultra-short-pulse-laser

## Abstract

The ultra-short-pulse (USP) laser joining of sapphire to iron is investigated by combining electron backscatter diffraction (EBSD) and ruby (Cr^3+^) R_1_ fluorescence mapping to resolve the joint microstructure and pressure distributions. Energy-dispersive X-ray spectroscopy (EDS) reveals Al, O, and Fe intermixing within the seam, consistent with the formation of thin Fe–Al–O reaction layers. R_1_ fluorescence yields a maximum internal pressure of (490±80) MPa within the modified sapphire region and decays to near-zero within a few micrometres distance from the seam. EBSD data suggest a single-crystal sapphire lattice with localized disorientation adjacent to the joint, whereas the iron foil remains polycrystalline with rolling-induced misorientation without additional weld-induced grain refinement. These results demonstrate that USP joining of sapphire to iron produces localized interfacial reaction zones, with confined pressure predominantly occurring within sapphire.

## 1. Introduction

Direct sapphire (Al_2_O_3_)-to-metal bonding enables epoxy-free, compact and hermetic packaging concepts for optical and photonic instruments, but achieving reliable joints is non-trivial for dissimilar materials. The difficulty originates from pronounced mismatches in thermal expansion and elastic properties and the brittle fracture behavior of sapphire [1,2]. Conventional joining routes (e.g., adhesives, solder/braze interlayers, or high-temperature bonding concepts) often require global heating and introduce foreign phases [3,4]. Despite these challenges, sapphire-to-metal bonding is of significant interest, because sapphire offers excellent chemical/corrosion resistance and high thermal robustness while maintaining high optical transmission from the ultraviolet/visible to the infrared [5,6]. In particular, for optical instruments, epoxy-based bonding can be problematic due to outgassing and contamination effects, motivating adhesive-free joining strategies [7].

Transparent-to-metal USP laser welding was established in 2008, when Ozeki et al. first demonstrated direct femtosecond-laser welding of glass to copper, achieving joint strengths exceeding 16 MPa [8]. Since then, multiple groups have extended the concept to a wide variety of transparent–metal material pairs and evaluated joint performance using mechanical testing and reliability-oriented protocols, including thermal cycling [9,10,11,12,13,14,15,16]. For sapphire–metal systems in particular, reported shear strengths span a broad range depending on surface preparation and contact conditions, from 11 MPa to 12 MPa for rough, non-optical-contact joining (Sa up to 2 μm) to 108 MPa for optimized roughness-controlled interfaces [17,18]. These developments motivate a focused investigation of sapphire–iron welding, combining technologically relevant material pairing with a close analysis of the weld seam by complementary microstructure and stress-mapping methods.

While pre-process, in-process, and post-process quality assessments of cw- and ns-pulsed laser welds are well established [19], a comparable framework for ultrashort pulse laser welding is not yet fully developed. The first approaches to in-process assessment were based on monitoring the plasma emission during processing [20] or evaluating Newton’s rings through the writing objective [21]. Pre-processing studies have shown that surface preparation can have a significant influence on joint quality [17,18], while post-process evaluations so far have relied mainly on mechanical strength testing [9,11,15]. In contrast, a spatially resolved microstructural and spectroscopic post-process characterization of USP weld seams, as presented in this work, remains largely unexplored.

In this study, the focus is placed on a close analysis of the weld seam and its immediate vicinity in USP sapphire-to-iron welding by combining SEM/EDS for interfacial chemistry, EBSD-based crystallographic/strain analysis in the opaque metal, and non-contact residual-stress mapping in sapphire via the pressure-induced shift in the ruby (Cr^3+^) R_1_ fluorescence line [22,23,24,25].

Together, these methods provide a spatially resolved structure–chemistry–stress picture of the weld seam. The results reveal a localized interfacial reaction zone with confined pressure predominantly occurring within sapphire, while the iron foil remains largely unaffected in terms of microstructure and residual stress. Although the present case study focuses on sapphire–iron, it introduces concepts and analysis strategies that are more broadly applicable to the laser joining of transparent materials (e.g., glass) to metals. These insights improve the understanding of the underlying mechanisms of USP joining and guide future efforts to optimize robust transparent-material-to-metal welding.

## 2. Materials and Methods

### 2.1. Welding Process and Materials

**Figure 1:**
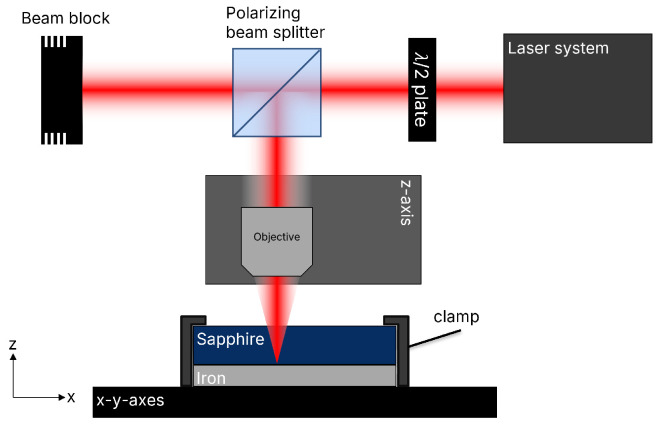
Schematic of the optical setup for laser processing. An ultrashort laser pulse is guided through a λ/2-wave plate and a polarization beam splitter. This is followed by the objective, which is mounted on a z-axis and determines the focus position on the workpiece. The sapphire–iron sample is fixed with a clamping device and can be moved using the mechanical x- and y-axes.

For the sapphire-to-metal welding process, we used an ultra-short pulse (USP) laser (Amplitude Tangor 50, France) in a setup shown in Figure 1. The laser system delivers pulses with durations in the range of 0.5–3.9 ps at a wavelength of 1030 nm, with adjustable repetition rates up to 40 MHz and a burst mode (25 ns pulse separation) operation. The raw beam diameter is 3.5 mm, and the laser power was precisely adjusted using a λ/2-wave plate and polarization beam splitter cube. The beam was directed through a dichroic mirror into a Mitutoyo objective (NA = 0.26).

Samples, consisting of sapphire substrates (SCHOTT AG, Mainz, Germany) and cold-rolled iron (Goodfellow, Hamburg, Germany), were brought into close contact using a custom clamping device. The material properties are listed in Table 1. This device maintained uniform mechanical pressure across the interface and was mounted on motorized X-Y translation stages, enabling precise three-dimensional positioning and controlled feed rates during welding. The process parameters were selected based on the Two-Threshold Criteria established in our previous work [21]. This framework identifies a low-fluence regime bounded by the intensity threshold for nonlinear absorption in the transparent material and the fluence threshold for ablation at the metal surface. The applied fluence of 0.35 J cm^−2^ is well below the reported single-pulse damage threshold of sapphire *F*_th_ = 11 J cm^−2^ [26], ensuring that nonlinear absorption and bulk modification in sapphire are strongly suppressed. A focus offset of $z_{f}=-100\;\mu m$ was chosen intentionally to produce a wider weld seam, facilitating spatially resolved microstructural and spectroscopic analysis of the weld cross-section. The process parameters are listed in Table 2.

### 2.2. Sapphire Pressure Measurement via Ruby Fluorescence

Residual stresses in the sapphire adjacent to the weld interface were quantified using fluorescence spectroscopy based on the ruby pressure scale. This method exploits the hydrostatic-pressure-induced shift in the $R_{1}$ fluorescence line in ${\mathrm{Cr}}^{3+}$-doped Al_2_O_3_ (ruby), which is a well-established technique for non-destructive pressure mapping. Fluorescence measurements were performed using a confocal Raman spectrometer (WiTec alpha 300R) equipped with a 532 nm excitation laser and a 100× objective (Zeiss, Jena, Germany). The $R_{1}$ fluorescence line was recorded in a raster scan across the weld region, with a spatial resolution of 1 μm and an integration time of five seconds per point. The peak position of the $R_{1}$ line was determined by fitting a double Lorentzian function to each spectrum. In the absence of any visible deviatoric component, which should be observable from R_1_–R_2_ peak splitting and broadening [27,28], the shift in the R_1_ fluorescence line can be assigned to a purely hydrostatic effect. The internal pressure was calculated using the IPPS-Ruby2020 calibration [24]:

(1) $$p\left(\lambda_{\mathrm{calc}}\right)\;\left[\mathrm{GPa}\right]=1.87\times 10^{3}\;\left(\frac{\lambda_{\mathrm{calc}}-\lambda_{0}}{\lambda_{0}}\right)\left(1+5.63\;\left(\frac{\lambda_{\mathrm{calc}}-\lambda_{0}}{\lambda_{0}}\right)\right)$$

All fluorescence measurements were performed post-process at room temperature. The reference wavelength $\lambda_{0}$ was determined on unmodified sapphire within the same sample, so that any systematic temperature offset is cancelled out in the pressure calculation. A detailed description of the measurement procedure and error analysis is provided in Appendix A.

### 2.3. Electron Backscatter Diffraction (EBSD) Analysis

To investigate the crystallographic structure and phase composition of the weld interface and adjacent regions, electron backscatter diffraction (EBSD) was performed on cross-sections of the welded samples. Specimens were first embedded in epoxy resin, then ground and polished to a final surface roughness of 1 μm using diamond suspensions. For EBSD, a final polishing step was carried out using a cross-section polisher with argon ion milling to minimize surface deformation and achieve optimal pattern quality. EBSD measurements were conducted using a field emission scanning electron microscope (SEM, ZEISS Gemini) equipped with an Oxford Instruments Symmetry S3 EBSD detector. The SEM was operated at an accelerating voltage of 20 kV and a probe current of 10 nA. The sample surface was tilted by 70° relative to the incident electron beam to maximize Kikuchi pattern quality. EBSD data were acquired and processed using the AZtec (version 6.1) software suite, with phase identification performed through comparison with crystallographic databases containing relevant phases (e.g., Fe, FeO, Fe_3_C, Al_2_O_3_, FeAl_2_O_4_). Grain orientation, phase distribution, and local misorientation were analyzed to assess microstructural changes, recrystallization, and residual strain in the weld region.

## 3. Results and Discussion

EDS and ruby fluorescence pressure mapping were performed on multiple weld seams with reproducible results. EBSD analysis was conducted on a single representative weld cross-section. All the results presented in the following are taken from the same cross-section to ensure direct comparability between the different characterization methods.

**Figure 2:**
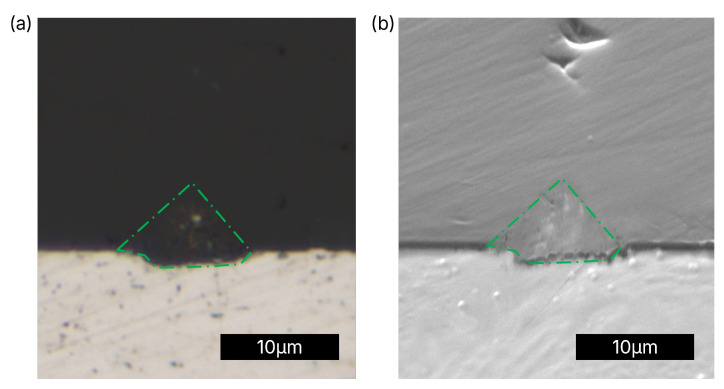
Micrograph (**a**) and scanning electron microscope image (**b**) of the cross-section of a sapphire-to-iron weld seam, which is indicated by the green dashed line. The weld seam is located at the interface and has a triangular shape on the sapphire side and reaches about 2 μm into the iron surface.

Optical microscopy of the sapphire–iron interface, as shown in Figure 2a, reveals a distinct separation between the sapphire substrate and the iron, with the weld seam appearing as a narrow, localized zone at the interface. At this scale, the internal structure of the seam cannot be resolved. The SEM imaging, as shown in Figure 2b, provides deeper insight into the weld seam morphology. Here, the seam exhibits a triangular geometry extending into the sapphire with a height up to 6 μm, while penetrating approximately 2 μm into the metal surface. Within the structure, no macroscopic cracks or voids are visible.

**Figure 3:**
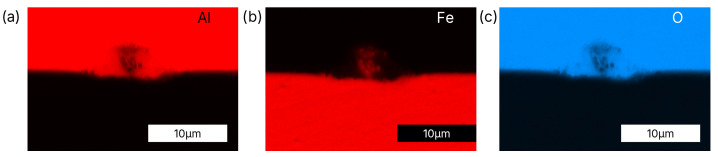
The image shows EDS analysis of the sapphire-to-iron cross-sections for (**a**) aluminum (**b**) iron and (**c**) oxygen. The distribution of the elements clearly shows that the zone in which the sapphire and iron mix is limited to the interior of the weld seam. It can also be seen that the main part of the weld seam consists of the components of the sapphire and that only a small amount of iron was mixed into the weld seam.

Energy-dispersive X-ray spectroscopy (EDS) was employed to further analyze the weld seam, resulting in elemental maps documenting the presence of oxygen, aluminum, and iron, as can be seen in Figure 3. EDS analysis shows that the weld contains a mixture of sapphire and iron phases. The inner part of the weld contains iron components that protrude into the sapphire material along the triangular structure. Furthermore, the sapphire components are seen to extend up to 2 µm into the iron surface. These two observations correspond to the weld structure shown in SEM in Figure 2b.

### 3.1. Pressure Mapping

**Figure 4:**
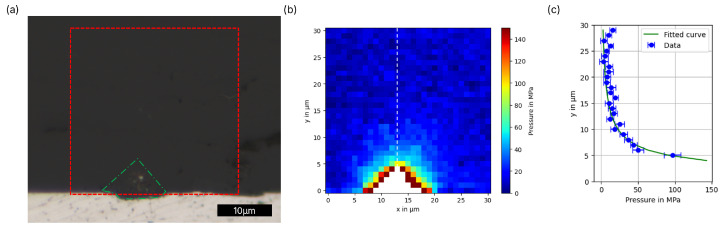
Visualization of the pressure in the sapphire in and around the weld seam. In (**a**), a micrograph of the sapphire iron interface is shown, with the measurement area marked by the red dotted square. The weld seam countour is indicated by a green dashed line. In subfigure (**b**), the calculated pressure in the measured area is given. For the points within the weld seam, no pressure can be determined due to insufficient signal, which is why the points are shown here as white. The maximum calculated pressure next to the weld seam is (490 ± 80) MPa. Since pressures above 150 MPa occur only in the immediate vicinity of the weld boundary, the color scale is capped at this value to resolve the spatial pressure gradient in the surrounding sapphire. In (**c**), the pressure distribution along the white dashed line in (**b**) is shown by a fitted curve according to Equation (2).

The polished cross-sections of the samples were analyzed using the pressure-sensitive $R_{1}$ fluorescence peak. An evaluation of the weld seam from the previous section can be seen in Figure 4. The area surrounding the weld seam that was analyzed is displayed. An increase in pressure is noticeable in the immediate vicinity of the weld. The maximum pressure value in the modified area directly adjacent to the weld is (490 ± 80) MPa. The pressure then decreases with increasing distance from the weld, dropping to 0 MPa after approximately 10 μm from the weld boundary. There is no significant measurable difference compared to the unmodified bulk material.

The pressure distribution along the central line (white dashed line in Figure 4b is shown in Figure 4c. The data are well-described by an experimental power-law decay of the form

(2) $$P\left(y\right)=\frac{a}{y^{2}}.$$

This analysis shows that, although the modification to the sapphire–metal interface is highly localized, the weld seam still has a measurable influence on the surrounding unmodified sapphire.

One possible cause of this pressure is that the laser-welding process, with its associated high temperatures in the plasma zone at the sapphire–metal interface, causes the sapphire to melt. During subsequent rapid cooling, the directly mixed melted sapphire cannot necessarily resolidify into the same well-organized state as the surrounding crystal. As a first-order elastic estimate, the measured peak pressure may be interpreted as a hydrostatic compression of the surrounding sapphire. Using the bulk modulus of sapphire (*K* = 240 × 10^3^ MPa), this corresponds to a volumetric strain on the order of

(3) $$\frac{\Delta V}{V}\approx \frac{\Delta p}{K}=\frac{490\;\mathrm{MPa}}{240\times 10^{3}\mathrm{MPa}}\approx 0.2\%.$$

The observed pressure field can be interpreted as the elastic response to a local volumetric mismatch between the modified interfacial zone and the surrounding single-crystal sapphire. Several mechanisms are expected to contribute to this mismatch. First, the resolidification of sapphire into a polycrystalline or partially disordered state within the weld seam results in a different density compared to the surrounding single-crystal lattice. Second, as discussed in the following section, new phases are formed within the weld seam that exhibit a different molar volume than the parent phases, adding to the local volumetric strain. With the present data, the individual contributions of these mechanisms cannot be separated quantitatively, but the measured pressure field is consistent with their combined effect. A finite-element analysis accounting for the actual weld geometry and the bimaterial interface would be required to fully model the origin of the observed hydrostatic stress field.

### 3.2. Phase Distribution and Crystal Lattice Deformation

**Figure 5:**
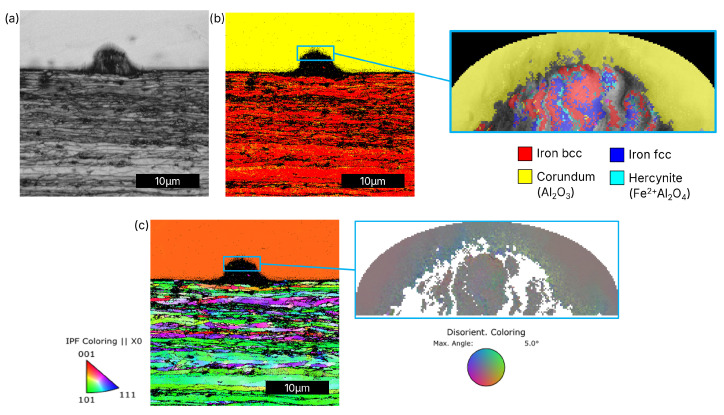
Result of the EBSD-analyzed welding area. In (**a**) the iron band contrast, in (**b**) the phase map, and in (**c**) the inverse pole figure (IPF) parallel to X of the sapphire-to-iron welding connection are shown. Local lattice disorientation within the upper, modified sapphire region is shown next to (**c**).

EBSD analysis was employed to identify formed phases within the weld seam and to investigate changes in the crystal lattice orientation in the vicinity of the weld seam as indicators of internal stress. The sapphire region exhibited a single-crystal structure, whereas the iron foil displayed a polycrystalline morphology characteristic of rolled material, as can be seen in Figure 5. In the iron substrate, grain misorientation of the elongated grains was observed throughout the bulk, reflecting its manufacturing history, but no additional orientation changes or grain refinement were detected in proximity to the weld, suggesting that the welding process did not induce measurable structural alterations in the metal. Conversely, the sapphire revealed localized lattice disorientation adjacent to the weld interface, visualized in the color-coded misorientation map in Figure 5c. This disorientation is strictly confined to the immediate vicinity of the weld seam, while the surrounding sapphire exhibits a uniform single-crystal orientation, serving as an internal reference for the unmodified state. EBSD data allow only limited phase identification, but they constrain the phases present within the weld seam and their spatial distribution.

This analysis indicates the partial transformation of iron from α-Fe (bcc) to γ-Fe (fcc), as well as the formation of hercynite (FeAl_2_O_4_), a spinel structure, as proof of an effective chemical reaction between sapphire and iron. At this α-Fe to γ-Fe transition, near 1184 K, the molar volume of iron decreases from 7.37 cm^3^ mol^−1^ to 7.30 cm^3^ mol^−1^, indicating a slight densification of about 1% [29]. This demonstrates the alternation between high- and low-density regions within the weld seam. Notably, the detection of hercynite is remarkable because this spinel phase is typically formed by oxidation-assisted interfacial reactions between Al_2_O_3_ and Fe–O species (e.g., FeO), indicating that chemical bonding reactions occurred at the sapphire–iron interface rather than a purely physical mixture.

In addition to identifying the bcc and fcc phases of iron, the EBSD phase map reveals a lack of chemical and structural homogeneity in the weld seam. It exhibits a vertically stratified phase distribution along the beam direction. Specifically, regions dominated by bcc iron appear to alternate with regions in which fcc iron and hercynite coexist. Ultrafast laser processing is known to generate self-organized periodic nanostructures in transparent materials [30,31], so it is possible that a related self-organization mechanism is at work in the present weld seam. One possible explanation for the observed phase layering is a transient spatial variation in iron and oxygen content within the laser interaction zone.

While the pressure analysis of the sapphire in the previous section revealed a measurable influence zone of the weld on the surrounding material, the EBSD analysis showed no deformation of the iron foil outside the weld. It cannot be fully excluded that small residual elastic strains exist in the iron below the detection limit of the EBSD measurement. However, the highly localized energy deposition characteristic of USP welding confines the heat-affected zone to the immediate weld region, which limits the spatial extent of any thermal stress in the metal. The absence of detectable orientation changes in the iron outside the weld seam is therefore consistent with the confined nature of the USP process.

## 4. Conclusions

This work demonstrates that ultra-short-pulse (USP) laser welding enables the direct joining of sapphire (Al_2_O_3_) to iron with a highly localized reaction zone and confined stress/pressure fields in the sapphire. Using spatially resolved ruby $R_{1}$ fluorescence mapping, internal pressures up to (490±80) MPa were quantified in the modified sapphire directly adjacent to the weld, decaying to near-zero within approximately 10 μm from the weld boundary. EBSD confirmed that the iron foil retains its rolling-induced polycrystalline microstructure without detectable weld-induced grain refinement or additional orientation changes outside the weld seam, whereas sapphire exhibits localized lattice disorientation near the interface. Within the seam, EBSD-based phase analysis indicates the partial recrystallization of iron from bcc to fcc and the presence of hercynite (FeAl_2_O_4_), suggesting oxidation-assisted compound formation at the sapphire–iron interface rather than a purely physical mixture. Overall, the combined structure–chemistry–pressure analysis enables localization of the weld-affected zone: structural changes are confined to the seam in the metal, while a measurable pressure field extends into sapphire over a micrometre-scale vicinity.

The presented ruby–fluorescence pressure mapping provides a quantitative, non-contact tool to systematically compare sapphire–metal joints in future studies. Combined with correlative microstructure and phase analysis, this can support the targeted optimization of laser parameters and metal-surface preparation to improve joint quality and reliability. These observations are consistent with the Two-Threshold Criteria established in our previous work, according to which operation in the low-fluence regime confines the interaction zone and limits residual-stress development, thereby reducing the tendency of crack formation.

Future work should include a high-resolution TEM analysis of the weld seam to independently confirm the hercynite phase assignment and to resolve the nanoscale structure of the interfacial reaction zone, complemented by nano-FTIR and micro-Raman spectroscopy to provide vibrational and chemical fingerprints of the phases formed during the welding process. Furthermore, establishing quantitative structure–property relationships that link the observed microstructural features and residual pressure fields to mechanical joint performance, such as shear strength and thermal cycling reliability, constitutes a natural extension of this work.

## Figures and Tables

**Table 1:** Material parameters of iron and sapphire. Symbols: *t* thickness (mm); CS crystal structure; $T_{m}$ melting temperature (°C); $c_{p}$ specific heat capacity (kJ kg^−1^ K^−1^) (at 25 °C); *k* thermal conductivity (W m^−1^ K^−1^) (at 25 °C); α coefficient of thermal expansion (10^−6^ K^−1^) (at 25 °C). For sapphire, $\alpha_{\|c}$ and $\alpha_{⊥c}$ denote values parallel and perpendicular to the crystallographic *c*-axis, respectively.

	*t*	CS	$T_{m}$	$c_{p}$	*k*	α
Iron	0.05	bcc	1535	0.444	80.4	11.8
Sapphire	1.75	hcp	2040	0.774	27.21	$\alpha_{\|c}=6.7$, $\alpha_{⊥c}=5$

**Table 2:** Process parameters used for the ultrashort-pulse welding process. Symbols: $E_{p}$ pulse energy (μJ); *f* repetition rate (kHz); $\tau_{p}$ pulse duration (ps); *N* number of pulses within a burst; *v* processing speed (mm s^−1^); $z_{f}$ focus position (μm); *d* beam diameter at the interface (μm); *F* fluence at the sapphire–iron interface (J cm^−2^).

$E_{p}$	*f*	$\tau_{p}$	*N*	*v*	$z_{f}$	*d*	*F*
0.43	500	3	4	5	−100	12.5	0.35

## Data Availability

The data presented in this study are available upon reasonable request from the corresponding author.

## Appendix A. Detailed Description of the Sapphire Pressure Measurement Method

For this method, every single spectrum of the measurement grid was analyzed through a standardized sequence of post-processing steps. First, the spectral data were cropped to a range from 4300 cm^−1^ to 4500 cm^−1^. Second, the spectra were despeckled using the Whitaker–Hayes algorithm to remove outlier spikes. Third, anchor points were set at 4300 cm^−1^ and 4500 cm^−1^ and a linear background subtraction was performed between these points. Fourth, the maximum intensity of each spectrum was scaled to 1 to facilitate direct comparison across all data points.

For every spectrum acquired during the fluorescence measurements, a double-Lorentzian function was fitted to accurately determine the peak position of the R1 line. The fitting function used is

(A1) $$I\left(\omega \right)=\frac{A_{1}}{1+{\left(\frac{\omega -\omega_{1}}{\Gamma_{1}}\right)}^{2}}+\frac{A_{2}}{1+{\left(\frac{\omega -\omega_{2}}{\Gamma_{2}}\right)}^{2}},$$

where I(ω) is the intensity at wavenumber ω, $A_{1}$ and $A_{2}$ are the amplitudes, $\omega_{1}$ and $\omega_{2}$ are the center positions of the two Lorentzian components, and $\Gamma_{1}$ and $\Gamma_{2}$ are the respective half-widths at half-maximum.

The determined center positions were used to calculate the corresponding wavelength via the conversion from wavenumber (in cm^−1^) to wavelength (in nm):

(A2) $$\lambda \left(\omega \right)=\frac{1}{\left(\frac{1}{\lambda_{\mathrm{Laser}}}-\frac{\omega }{10^{7}}\right)},$$

where $\lambda_{\mathrm{Laser}}=532\;nm$.

The pressure was calculated using the calibration of Shen et al. (2020) [24]:

(A3) $$p\left(\lambda_{\mathrm{calc}}\right)=1.87\times 10^{3}\left(\frac{\lambda_{\mathrm{calc}}-\lambda_{0}}{\lambda_{0}}\right)\left[1+5.63\left(\frac{\lambda_{\mathrm{calc}}-\lambda_{0}}{\lambda_{0}}\right)\right],$$

where *p* denotes the pressure in GPa, $\lambda_{\mathrm{calc}}$ is the wavelength calculated from the shifted spectral line, and $\lambda_{0}$ is the unshifted R_1_ reference wavelength. To calibrate the system, an unmodified area within the sapphire sample was measured. With the given setup, the central wavelength without shift was

$$\lambda_{0}=(694.252\pm 0.001)\;nm\;\left(\omega_{1}=(4393.0060\pm 0.0234){\mathrm{cm}}^{-1}\right).$$

To ensure data quality, a masking procedure was applied to exclude erroneous measurements. A data point was discarded if both conditions were met simultaneously:

(A4) $$\left|\Delta p\right|\;>\;0.01\;\mathrm{GPa}\;\mathrm{and}\;\frac{\left|\Delta p\right|}{\left|p\right|}>0.2.$$

This dual-condition masking ensures that only high-confidence measurements contribute to the final map.
